# Specific Alu elements involved in a significant percentage of copy number variations of the *STK11* gene in patients with Peutz–Jeghers syndrome

**DOI:** 10.1007/s10689-015-9800-5

**Published:** 2015-04-05

**Authors:** Pawel Borun, Marina De Rosa, Boguslaw Nedoszytko, Jaroslaw Walkowiak, Andrzej Plawski

**Affiliations:** 1 Institute of Human Genetics, Polish Academy of Science, Strzeszynska 32, 60-479 Poznan, Poland; 2 Department of Molecular Medicine and Medical Biotechnology and CEINGE, University of Naples “Federico II”, Naples, Italy; 3 Department of Dermatology, Venereology and Allergology, Medical University of Gdansk, Gdansk, Poland; 4 Department of Pediatric Gastroenterology and Metabolic Diseases, University of Medical Sciences, Poznan, Poland

**Keywords:** Alu elements, Copy number variation, CNV, PJS, Peutz–Jeghers syndrome, *STK11*

## Abstract

Peutz–Jeghers syndrome (PJS) is a rare hereditary syndrome characterized by the occurrence of hamartomatous polyps in the gastrointestinal tract, mucocutaneous pigmentation and increased risk of cancer in multiple internal organs. PJS is preconditioned by the manifestation of mutations in the *STK11* gene. The majority of detected *STK11* changes are small scale mutations, however recent studies showed the significant contribution of medium-sized changes commonly known as copy number variations (CNVs). Here we present a novel 7001 bps deletion of *STK11* gene fragment, in which we identified the presence of breakpoints (BPs) within the Alu elements. Comparative meta-analysis with the 80 other CNV cases from 12 publications describing *STK11* mutations in patients with PJS revealed the participation of specific Alu elements in all deletions of exons 2–3 so far described. Moreover, we have shown their involvement in the two other CNVs, deletion of exon 2 and deletion of exon 1–3 respectively. Deletion of exons 2–3 of the *STK11* gene may prove to be the most recurrent large rearrangement causing PJS. In addition, the sequences present in its BPs may be involved in a formation of a significant percentage of the remaining gene CNVs. This gives a new insight into the conditioning of this rare disease and enables improvements in PJS genetic diagnostics.

## Introduction

Peutz–Jeghers syndrome (PJS; OMIM 175200) is a predisposition to the occurrence of pigment skin lesions and hamartomatous polyps inherited in an autosomal dominant way. The polyps may cause ileus and bleeding from the lower part of the tract, if underwent autoamputation [1, 2]. Incidence of the syndrome ranges from 1/29,000 to 1/120,000 births [3]. Patients with PJS are at an elevated risk of developing extra intestinal cancers located in the pancreas, the breasts, the ovary and the uterus [4–6].

Peutz–Jeghers syndrome is preconditioned by the manifestation of mutations in the *STK11* (OMIM*602216 Serine/Threonine Protein Kinase 11) gene, located in the short arm of chromosome 19, in the 13.3 region. The gene consists of ten exons, nine of which code for a protein. The majority of detected changes are small scale mutations, however recent studies showed the contribution of large rearrangements in a significant percentage of patients, reaching in some groups even 30 % of all the detected mutations [7, 8]. These medium-sized changes concerning exons or larger genomic fragments are commonly known as copy number variations (CNV) [9]. Literature indicates a number of mechanisms involved in the formation of CNV [10]. One of the hypotheses described assumes that a significant proportion of large mutations occurs within the interspersed repeats through non-allelic homologous recombination. Alu elements are a family of short interspersed nuclear elements (SINE) and comprise about 10.5 % of the human genome [11]. There is an overrepresentation of such sequences in the *STK11* gene [12]. Alu elements, which represent 19 % of the entire gene sequence (24 kb), are concentrated mainly in the distal part of the gene (especially in intron 1). In the following publication we present the case of a patient with a deletion of *STK11* gene fragment, in which we observed the presence of breakpoints (BPs) within the Alu elements. We decided to perform a meta-analysis of similar cases reported in the literature, using the in silico analysis of the DNA sequence of the *STK11* gene.

## Materials and methods

### PJS family description

The proband is a 39 year-old woman (born in 1975), who manifested pigmented lesions of the lips and buccal mucosa during infancy. In preschool and school age pigmentation increased, appearing around the mouth, on the eyelids and around fingers. After puberty these signs gradually subsided. Currently 39, she exhibits small muco-cutaneous melanosis around the lips and eyelids. From the age of 21 the patient has undergone surgery four times to remove polyps from the stomach, small intestine and colon, preceded by signs of mechanical obstruction and gastrointestinal bleeding. All removed polyps were hamartomas with no signs of malignant transformation.

The proband’s mother also had pigmented lesions on the lips and buccal mucosa. She underwent surgery twice (in 1985 and 2000) because of gastrointestinal polyps. All observed polyps were also hamartomas with no signs of malignant transformation. The proband’s grandmother died from skin cancer of right groin, diagnosed as a melanoma while her grandfather died aged 54 due to “twisted bowel” (lack of precise clinical data). PJS in the family has been also diagnosed in the proband’s two children, sister and nephew.

### Study design

The material for research was DNA from peripheral blood. The blood was collected in cooperation with the Department of Dermatology of Gdansk Medical University. The studies were approved by the local Ethics Committee of the Poznan University of Medical Science and performed after obtaining written informed consent from the patients. The preliminary diagnostics using high resolution melting (HRM) and single strand conformation polymorphism (SSCP) showed no small mutations [13]. Subsequently, the patient was examined for large rearrangements using multiplex ligation-dependent probe amplification (MLPA) method (SALSA MLPA kit P101-A2 [MRC Holland]), which revealed decreased signals for probes hybridizing to exons 2 and 3.

Location of interspersed repeats within the entire sequence (24 kbps) of the *STK11* gene was determined on the basis of bioinformatic analysis with the use of RepeatMasker software, version open-3.3.0 (www.repeatmasker.org).

Primer pair encompassing potentially involved Alu sequences within introns 1 and 3 was designed to identify the exact location of mutation breakpoints. Primer sequences were as follows: forward—5′-TTCTGGTAGGCAGTGGGTTC-3′; reverse—5′-TCACAGAAGTCCAGGCACAC-3′. The 8936 bps long PCR product was obtained through Long-range PCR using Advantage Genomic LA Polymerase [Clontech]. Long-range PCR conditions were as follows: 94 °C for 1 min, and then 30 cycles of 94 °C for 30 s, and 68 °C for 13.5 min, and finally 72 °C for 10 min. The 25-μL reaction volume contained 100 ng of DNA and 5 pmol of each primer. PCR products were separated on 1 % agarose gel [Sigma Aldrich] electrophoresis in 1x TBE buffer. The shorter (approximately 2000 bps) product was directly sequenced by Sanger method using ABI 373 DNA sequencer [Applied Biosystems] according to manufacturer’s instructions.

Interspersed repeats alignment was conducted with the use of basic local alignment search tool (BLAST) (http://blast.ncbi.nlm.nih.gov/Blast.cgi) optimized for somewhat similar sequences (blastn). Sequence nomenclature used to describe the breakpoints and interspersed repeats positions was based on the UCSC Genome Browser Feb. 2009 (GRCh37/hg19). The nomenclature of all previously described CNVs was converted for the purpose of publication in accordance with the above-mentioned guidelines (Tables 1, 2).

**Table 1:** Summary of the *STK11* gene exons 2–3 deletions in other populations

Study	Original mutation description	Mutation size	Distal BP (5′)	Proximal BP (3′)	Interspersed repeat at 5′ BP	Interspersed repeat at 3′ BP
Our study	c.280 + 5594_464 + 384del7001	7001	1,212,796	1,219,796	AluY(1)	AluY(3)
Chile [14]	1,213,168–1,219,550	6383 (6364 according to the authors)	1,213,168	1,219,550	AluY(2)	AluY(3)
Italy II [15]	1,164,146–1,164,152 1,170,695–1,170,701	6549	1,213,146–1,213,152	1,219,695–1,219,701	AluY(2)	AluY(3)
Italy II [15]	1,164,240–1,164,248 1,170,788–1,170,796	6548	1,213,240–1,213,248	1,219,788–1,219,796	AluY(2)	AluY(3)
Italy I [12]	NC_000019.8:g.6998_13998del (7 KB del. spanning exons 2–3) p.E98_G115del	7000	1,212,795	1,219,795	AluY(1)	AluY(3)
Hungary [16]	c.291-5484_464 + 384del6865	6865	1,212,932	1,219,796	FLAM_C (between AluY(1) and AluY(2)	AluY(3)
Germany [7]	c.291-?_464 + ?del	No data	No data	No data	No data	No data
Korea [17]	–	No data	No data	No data	No data	No data
UK [18]	c.333-?_424 + ?del	No data	No data	No data	No data	No data
Netherlands [19]	–	No data	No data	No data	No data	No data
Australia [8]	–	No data	No data	No data	No data	No data
Australia [8]	–	No data	No data	No data	No data	No data

**Table 2:** Summary of other CNVs with the involvement of Alu elements of interest in the *STK11* gene

Study	Original mutation description	Deleted exons	Mutation size	Distal BP (5′)	Proximal BP (3′)	Interspersed repeat at 5′ BP	Interspersed repeat at 3′ BP
Italy II [15]	1,155,146–1,155,173 1,170,719–1,170,746	Exon 1–3	15,572	1,204,146–1,204,173	1,219,719–1,219,746	AluSq	AluY(3)
Chile [14]	1,213,086–1,218,902	Exon 2	5816	1,213,086	1,218,902	AluY(2)	None

### Meta-analysis

We performed a comparative analysis with the 80 CNV cases of the *STK11* gene from 12 publications describing *STK11* mutations in patients with PJS [7, 8, 12, 14–22].

## Results

### Breakpoint identification

Using the MLPA and sequencing we identified a 7001 bps deletion c.280 + 5594_464 + 384del7001 co-segregating with PJS phenotype in this family.

The analysis of the *STK11* gene sequence using RepeatMasker software revealed the presence of 20 interspersed repeats, including 18 SINE/Alu and 2 LINE1 elements. Comparing the data from bioinformatic analysis of the mutation sequence and the interspersed repeats within the *STK11* gene, we found that both mutation breakpoints are located in Alu elements from AluY subfamily, in regions [1,212,558–1,212,842] and [1,219,530–1,219,843] respectively.

### In silico analysis results

**Fig. 1:**
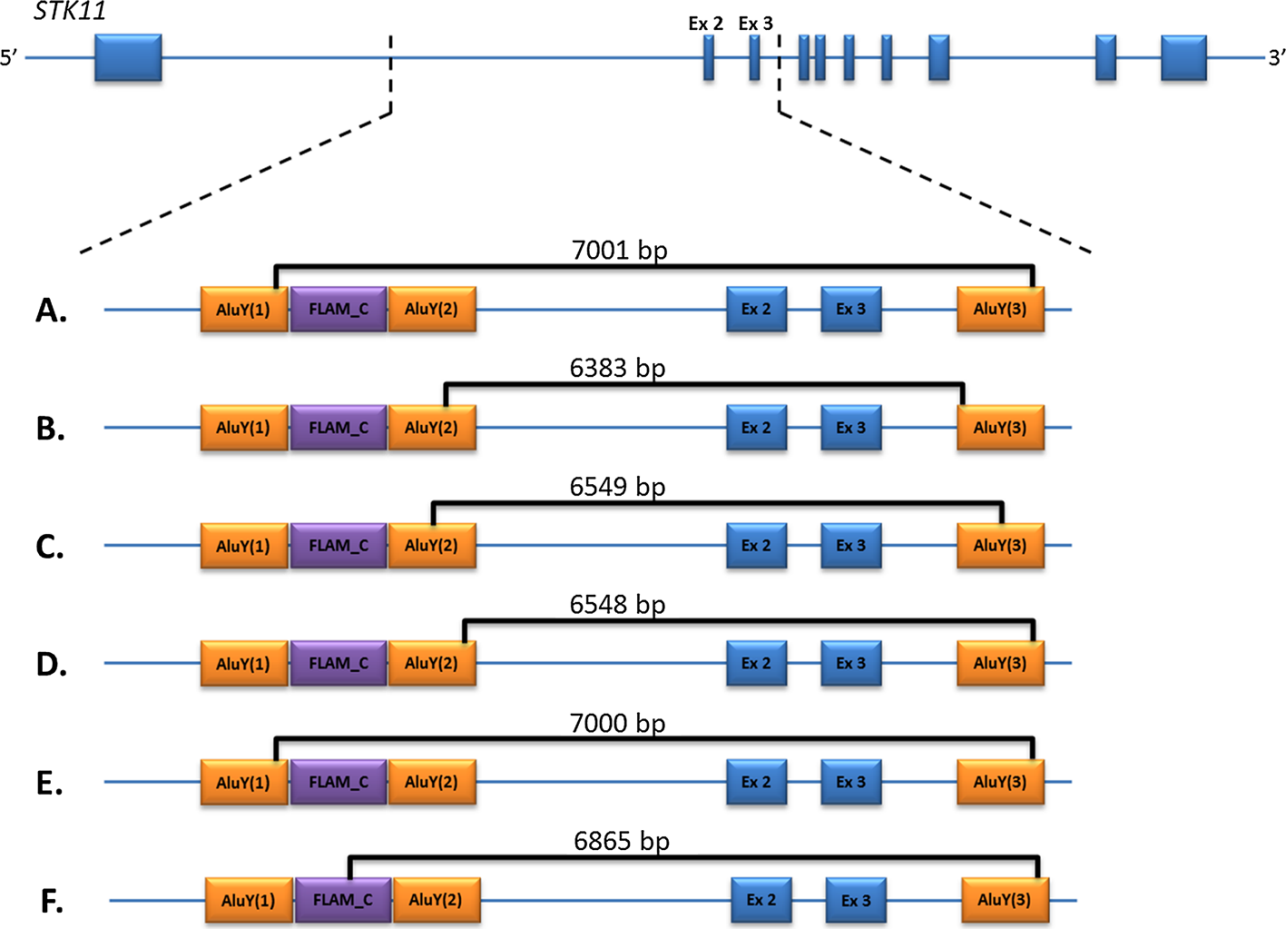
Approximate location of each *STK11* exons 2–3 deletion with identified breakpoint positions. Mutation order corresponds to the description in Table 1

Deletion of exons 2 and 3 of the *STK11* gene were observed also in PJS patients from other populations (Table 1). In several studies the exact breakpoint locations of detected CNVs were determined, and the results indicate the involvement of the same regions in the formation of all detected deletions of exons 2 and 3 (Fig. 1).

For distal breakpoints (5′) the participating region contains three interspersed repeats next to each other (all from class SINE and Alu family), respectively: AluY comprising sequence [1,212,558–1,212,842] [for the sake of publication it is referred to as AluY(1)], FLAM_C comprising sequence [1,212,850–1,212,978], and second AluY comprising sequence [1,212,891–1,213,195] [referred to as AluY(2)]. All proximal breakpoints (3′) are within the same Alu element from AluY subfamily comprising sequence [1,219,530–1,219,843] [referred to as AluY(3)].

Subsequently, we performed an alignment of AluY(3) sequence with the elements from 5′ BP and all other interspersed repeats within the gene located upstream (5′) from AluY (3). The analysis showed a high complementarity of AluY(3) to AluY(1)—232/295 (79 %), FLAM_C—84/112 (75 %) and AluY(2)—272/305 (89 %) and the position of all the above mentioned elements in the same orientation.

### Summary of clinical data

The phenotype of deletion carriers is generally typical for PJS (Table 3). All probands except one (diagnosed at the age of 1) had a characteristic pigmentation and hamartomatous polyps. Polyps in the stomach were observed in 25 % of the cases, and a single proband had nasal polyps. The age of diagnosis is varied. The course of disease in this group is not particularly severe. The available data show that among the probands occurrence of cancer has not been observed. In our proband’s family, melanoma occurred only in her grandmother.

**Table 3:** Summary of clinical data of the *STK11* exons 2–3 deletion carriers

Patient	Gender	Age at diagnosis	Pigmentation	Hamartomatous polyps	Polyps localization	Cancer
Our study	F	21	Yes	Yes	Stomach, small intestine, colon	No
Chile [14]	F	34	Yes	Yes	Small intestine, colon	No
Italy II [15]	M	4	Yes	Yes	Stomach, small intestine, colon	No
Italy II [15]	M	1	No	Yes	Stomach, small intestine, colon	No
Italy I [12]	N/A	5	Yes	Yes	Intestine	N/A
Hungary [16]	F	29	Yes	Yes	Small intestine	N/A
Germany [7]	N/A	N/A	Yes	Yes	N/A	N/A
Korea [17]	N/A	N/A	N/A	Yes	N/A	N/A
UK [18]	M	15	N/A	N/A	N/A	No
Netherlands [19]	N/A	N/A	N/A	Yes	Intestine, nasal cavity	N/A
Australia [8]	F	13	Yes	Yes	N/A	N/A
Australia [8]	F	35	Yes	Yes	N/A	N/A

*N/A* data not available, *F* female, *M* male

## Discussion

Molecular studies of genetic diseases rely on methods able to detect both small sequence variants (point mutations, etc.) and rearrangements of larger genomic fragments. Screening methods, along with direct sequencing, can identify the majority of small mutations. Copy number variations are a greater challenge and the methods used for their detection do not always allow researchers to determine the exact sequence undergoing mutation (e.g. MLPA, Quantitative-PCR, Array-CGH, C-HRM) [23–26]. These approaches not only fail to identify the exact breakpoint but also prevent any further examination of possible mechanisms involved in their formation.

**Fig. 2:**
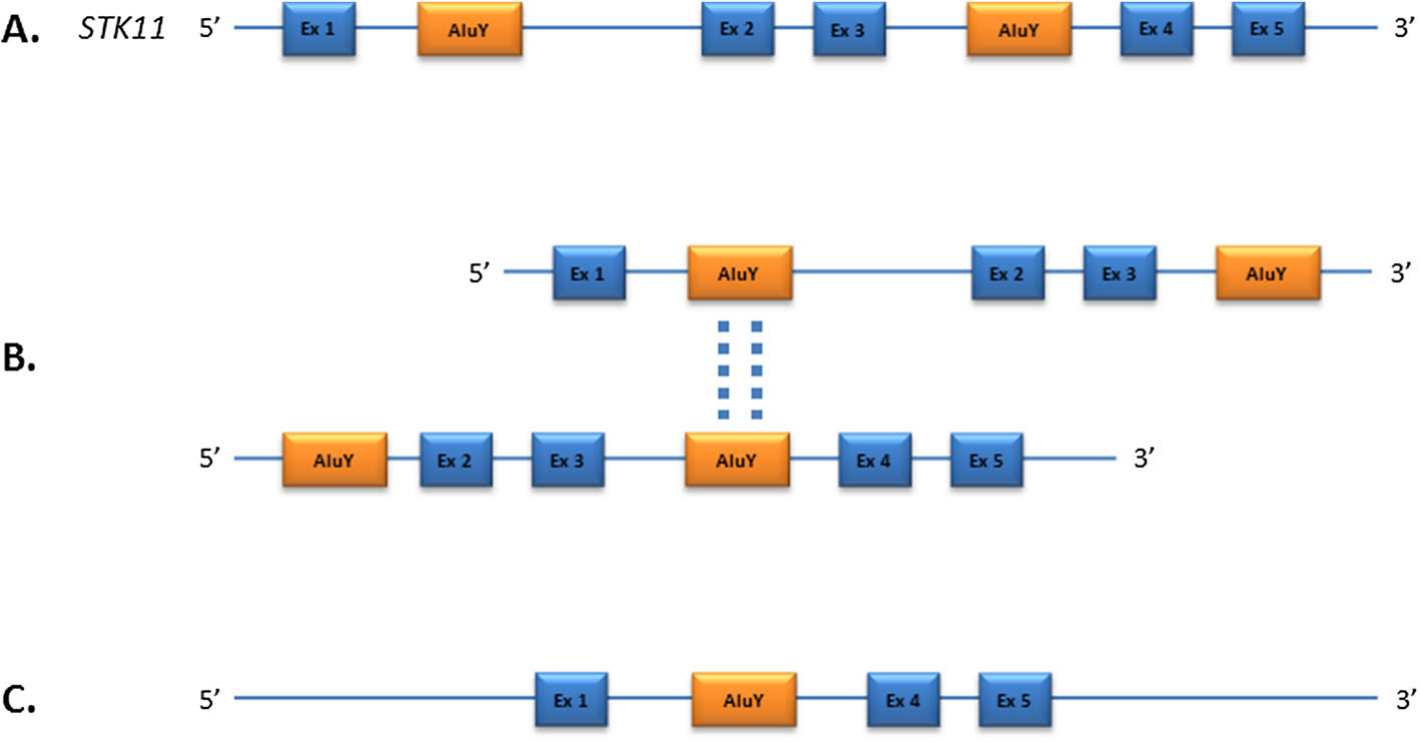
Possible CNV formation based on Alu-mediated homologous recombination model. **a** *STK11* gene structure, **b** recombination event between two homologous Alu sequences and **c** gene structure after recombination

In our study we have detected deletion of exons 2–3 of the STK11 gene in a family with PJS and determined the breakpoints of the mutation. Similar mutations had been described in studies of PJS patients from other populations. Among them 11 were deletions encompassing exons 2 and 3. Exact breakpoints were identified in five cases only, while the remaining ones provided no information on the size and exact location of the change. All six cases described (including our patient) are similar in terms of both BP positions, 3′ BPs in particular, which in all cases are within the AluY(3) element. All distal breakpoints (5′) are also located in the same region, with the difference that in silico analysis revealed the presence of three contiguous interspersed repeats: AluY(1), FLAM_C and AluY(2), respectively. All the above elements belong to the SINE class and Alu family. Although 5′ BPs of changes are not exactly in the same element, it can be assumed that the whole region is involved in the formation of CNV, and the BP position is merely the result of non-specific DNA repair. Involvement of Alu elements in all mutations analyzed may confirm De Rosa’s hypothesis that non-allelic homologous recombination mediated by Alu elements is a factor causing the formation of deletions of exons 2–3 in the *STK11* gene [12]. Moreover, this mechanism seems to be recurrent (Fig. 2).

Then we checked the complementarity between AluY(3) and other interspersed repeats present in the gene upstream from AluY(3). Sequence alignment showed high complementarity of AluY(3) to all elements from the distal BPs of analyzed changes. Furthermore, distal as well as proximal BPs are situated in the same orientation, which may be crucial for the mechanism involved in their formation.

**Fig. 3:**
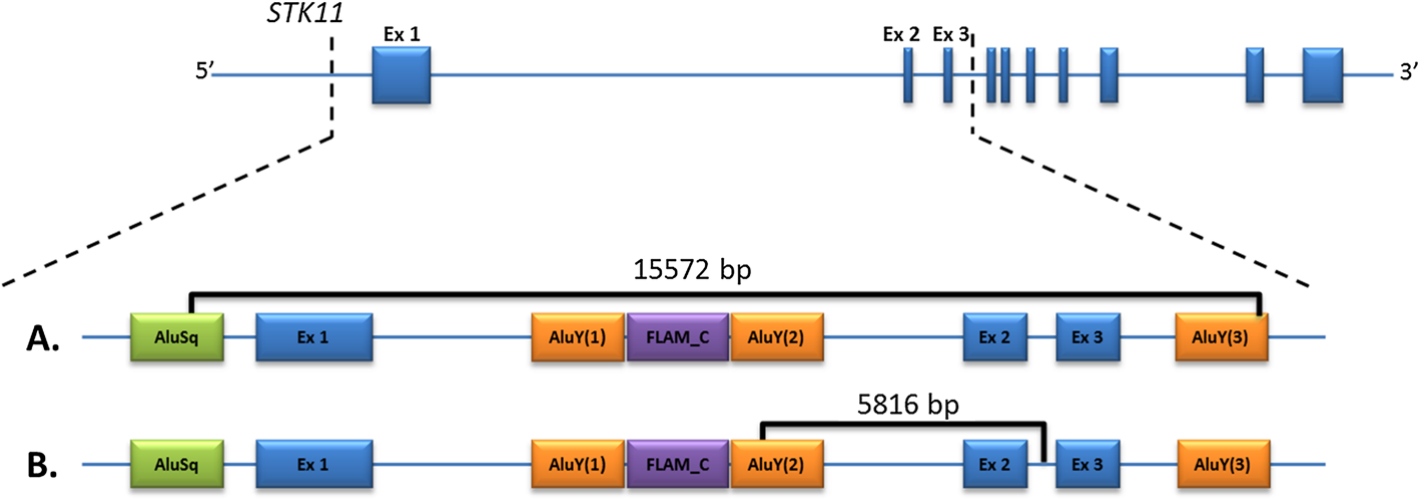
Approximate location of other CNVs with breakpoints present in the Alu elements of interest. Mutation order corresponds to the description in Table 2

Of the 80 CNVs described in publications we analyzed only 20 having determined breakpoints. Excluding five deletions of exons 2–3, BPs in CNVs encompassing this region were determined only for two changes that are: deletion of exon 2 described in Chile and deletion of exons 1–3 in Italy (Table 2). Both these changes share one BP with deletions of exons 2–3. Distal BP of exon 2 deletion is within AluY(2), and proximal BP of exons 1–3 deletion is in AluY(3) (Fig. 3). Of the total 21 CNVs with determined BPs, 8 (over 38 %) exhibit participation of Alu elements from these two regions.

Further analysis of the remaining 60 CNVs without specified BPs showed that there are 13 additional changes in which at least one BP is located in intron 1 or 3. It cannot be excluded that in their formation Alu elements of interest are involved. Of the 81 identified CNVs, 8 have confirmed BPs in these regions, while the other 19 are changes with their potential involvement. Hence, there are 27 conceivable changes from 81 CNVs, which represent over 33 % of all detected CNVs of the *STK11* gene.

The increased prevalence of CNV in the gene can be associated with the accumulation of interspersed repeats including Alu, especially in the distal region, which has already been observed and thoroughly discussed by Resta et al. [15]. Of the 15 patients with CNV in their group, 7 had deletions caused by NAHR between Alu elements. They defined an 18,019 bp-long region of the *STK11* gene as a recombination hotspot. Resta et al. indicated that the repetitive elements have been implicated in chromosomal aberrations because their presence increases instability of the region. Our observations confirm this thesis, furthermore narrow recombination hotspot for deletions of exons 2 and 3 to two regions, to the first with contiguously grouped AluY(1), FLAM_C, AluY(2) in intron 1, and to the second with a single copy of AluY(3) in intron 3.

Although Resta et al. consider the high density of Alu elements as an important factor, they pointed out that it does not seem sufficient to explain the high level of NAHR events since several genes with high Alu content such as thymidine kinase or beta-tubulin are not characterized by a high incidence of this type of damage. They mention that characteristic DNA motifs or additional features like high GC content may contribute to the generation of recurrent rearrangements of the *STK11* gene. In our opinion, besides aforementioned features, DNA methylation level may be considered as another related factor. A few studies showed the association between methylation status of repeat-rich regions and the occurrence of recombination (cross over) events [27–29]. Those observations, which indicate that hypermethylation may inhibit homologous recombination, might explain the participation of specific Alus, despite the presence of closer situated and highly homologous elements.

Concentration of CNV in the distal part of the gene led us to the hypothesis of possible participation of intra-intronic rearrangements of *STK11* intron 1 in conditioning PJS, in some cases with so far undetected genetic basis of the disease. This 11,213-bp long fragment is located in the *STK11* recombination hotspot and characterized by the highest accumulation of interspersed repeats within the gene, reaching 43 % of its sequence (GC content 58 %). It cannot be excluded that those rearrangements (either deletions, duplications, or e.g. inversions) within the intron, might interfere in the correct splicing or expression of *STK11* thereby conditioning PJS.

Deletion of exons 2–3 of the *STK11* gene may prove to be the most recurrent large rearrangement causing PJS. Furthermore, its prevalence is not restricted to a particular geographical region, but concerns the worldwide population. In addition, the sequences present in its BPs may be involved in a significant percentage of the remaining gene CNVs. It is rather clear that there are multiple mechanisms of CNVs formation, which include both recombination and replication-based mechanisms. Thus, as reported, copy number changes are not randomly distributed, but multiple genomic features can affect the probability of their occurrence [10]. The data currently available unambiguously suggest participation of Alu elements from the two studied regions in all deletions of exons 2–3 so far described, and we have also shown their involvement in the other two mutations. Literature data indicate that approximately 30 % of PJS cases are caused by CNVs. According to our study, a third involve Alu elements of interest, thus we can assume that over 10 % of all PJS cases are related to the mutations concerning those two regions. This gives a new insight into the conditioning of this rare disease and allows to introduce improvements in genetic diagnostics of the patients.
